# Validity of LupusQoL-China for the Assessment of Health Related Quality of Life in Chinese Patients with Systemic Lupus Erythematosus

**DOI:** 10.1371/journal.pone.0063795

**Published:** 2013-05-23

**Authors:** Su-li Wang, Bin Wu, Lin Leng, Richard Bucala, Liang-jing Lu

**Affiliations:** 1 Department of Rheumatology, Ren Ji Hospital, School of Medicine, Shanghai Jiao Tong University, Shanghai, China; 2 Clinical Outcomes and Economics Group, Department of Pharmacy, Ren Ji Hospital, School of Medicine, Shanghai Jiao Tong University, Shanghai, China; 3 Section of Rheumatology, Department of Medicine, Yale University School of Medicine, The Anlyan Center, New Haven, Connecticut, United States of America; Beth Israel Deaconess Medical Center, United States of America

## Abstract

**Objectives:**

To adapt and assess the validity and reliability of LupusQoL for use in Chinese patients with systemic lupus erythematosus (SLE).

**Methods:**

Debriefing interviews of subjects with SLE guided the language modifications of the tool. The process of adaptation proceeded according to the guideline and pre-testing results of LupusQoL-China. 220 SLE patients completed LupusQoL-China and a generic preference-based measurement of health EuroQoL scale (EQ-5D), and 20 patients repeated them after 2 weeks. Internal consistency (ICR) and test-retest (TRT) reliability, convergent and discriminant validity were examined. Factor analysis and Rasch analysis were performed.

**Results:**

The mean (SD) age of the 208 subjects with SLE was 33.93 (±9.19) years. ICR and TRT of the eight domains ranged from 0.811 to 0.965 and 0.836 to 0.974, respectively. The LupusQoL-China domains demonstrated substantial evidence of construct validity when compared with equivalent domains on the EQ-5D (physical health and usual activities r = −0.63, pain and pain/discomfort r = −0.778, emotional health and anxiety/depression r = −0.761, planning and usual activities r = −0.560). Most LupusQoL-China domains could discriminate patients with varied disease activities and end-organ damage (according to SELENA-SLEDAI and SLICC-DI). The principal component analysis revealed six factors, and confirmatory factor analysis result of which is similar to eight factors model.

**Conclusions:**

These results provide evidence that the LupusQoL-China is valid as a disease-specific HRQoL assessment tool for Chinese patients with SLE.

## Introduction

Systemic lupus erythematosus (SLE) is a chronic progressive autoimmune disease, which can affect any organ, including the skin, kidney, lung, brain, heart, and joints. In addition to natural disease progression, the long-term administration of glucocorticoids and other immunosuppressants may significantly impact patients' life quality. With progress in medical care, the 5-year survival rate for patients with SLE currently exceeds 90% [1]–[3]. A comprehensive objective assessment of the long-term outcomes of patients with SLE, including disease activity, accumulated tissue damage, and health-related quality of life (HRQoL) is desirable. A HRQoL assessment that evaluates patients’ health status and personal sense of well being may more accurately reflect the burden of SLE disease among patients. Therefore accurate HRQoL assessment of patients with SLE is desirable and draws increasingly attention.

It’s known that HRQoL among patients with SLE is worse than the general population, particularly those fall victim at an earlier age [4]. Prior studies of HRQoL in SLE have employed generic tools, such as the Short Form-36 (SF-36) [5], EuroQoL scale (EQ-5D) [6], or 20-Item Short Form Health Survey (SF-20) [7], however it was found that generic tools were not sensitive enough, and lacked of specific domains and items for SLE. Therefore, attention has been turned toward developing a more disease-specific questionnaire. LupusQoL, a new SLE-specific HRQoL measure, have been demonstrated to be applicable to SLE patients in the UK and USA [8], [9]. To our knowledge, no SLE-specific HRQoL tools have been validated in Mainland Chinese. According to official website of LupusQoL(http://lupusqol.com/), a Chinese version(Traditional Chinese) has been applied to application in Taiwan. Although the language is similar, there are meaningful differences between China Mainland and Taiwan in culture, economics, education, religion, medical environment, and healthcare systems. Due to higher levels of economic development and health administration, most SLE patients in Taiwan could receive standardized treatment at an early stage, and the disease could be controlled at a satisfactory level for a long time [10]. For this reason, HRQoL of SLE Taiwanese in physical, functional and social domains could be relatively better than Mainlanders. Therefore, the HRQoL of SLE patients in Mainland China may benefit from specific differences from Taiwan. Our objective was to cross-culturally adapt the LupusQoL to Mainland Chinese, and evaluate its measurement properties.

## Patients and Methods

### Demographic Data

This study was approved by the institutional review board of Shanghai Jiaotong University, and all subjects consented to participation. We included consecutive SLE patients (n = 220) who had been followed up at Ren Ji Hospital, School of Medicine, Shanghai Jiao Tong University from March to November of 2012. The inclusion criteria were as follows: age between 18 and 75 years, and diagnosis of SLE according to the 1997 modified ACR criteria [11]. Demographic information included sex, age, age at disease diagnosis, education and marital status.

### LupusQoL

LupusQoL is a lupus-specific HRQOL questionnaire consisting of 34 items grouped in eight domains: physical health (PH), pain (PN), planning (PL), intimate relationships (IR), burden to others (BU), emotional health (EH), body image (BI) and fatigue (F) [8]. It has a five-point Likert response format, where 4 = never, 3 = occasionally, 2 = a good bit of the time, 1 = most of the time, and 0 = all of the time. Modifications made between the Taiwanese and Mainland Chinese version of LupusQol are described in the *Results*. Summary scores for LupusQoL-China were calculated guided by the scoring guidelines for LupusQoL-UK [8].

### EuroQoL Scale (EQ-5D)

The EQ-5D is a generic preference-based measurement of health [[12]. It includes a health state classifier that consists of five dimensions: mobility, self-care, usual activities, pain/discomfort and anxiety/depression. A utility index-based summary score is derived from the self-classifier, where 1 represents full health and 0 represents dead. Scores for the five domains of the EQ-5D were generated.

### Disease Characteristics

Disease activity was determined using the SLE Disease Activity Index (SLEDAI) instrument [13]. The SLEDAI is a 24-item instrument for assessing SLE activity in nine organ systems. Clinical and laboratory data are required to complete the questionnaire. The score ranges from 0 to 105 points, with higher values signifying greater disease activity. Damage was assessed using the SLE damage index (SLICC-ACR) tool [14]. SLICC/ACR-DI tracks irreversible organ dysfunction across 12 systems. The dysfunction must be present for 6 consecutive months in order to register as damage. The score ranges from 0 to 46, with higher scores signifying more damage.

### Statistical Analyses

We used SPSS software, version 10.0 to analyze data. Descriptive statistics were reported. The continuous variables were tested for normality; a non-parametric test (Mann-Whitney) was used for comparing continuous data.

### Content Validity

As LupusQoL was modified from a pre-existing Taiwan version, it was assumed that it had a good content validity. We asked the pre-test sample of patients whether the items were understandable, and made minor changes to the expression according the feedback.

### Internal Consistency Reliability

Cronbach’s α was used to test for internal consistency, and was considered satisfactory when ≥0.7 [15].

### Test-retest Reliability

Test-retest reliability was determined using an intraclass coefficient to assess the stability of the measure, comparing LupusQoL-China domains scores at baseline and 2 weeks later in patients (n = 20) whose self-assessed quality of life was rated as no change on a 15-point health status change scale (−7 to +7).

### Discriminant Validity

Discriminant validity is used to assess whether the instrument can distinguish between patients of different disease severity. There is not a validated instrument for severity of SLE, so we used both disease activity and damage to define disease severity, which were determined by SLEDAI and SLICC-DI. It was hypothesized that LupusQoL domains would be significantly altered in patients with a SELENA-SLEDAI score cutoff of 4 or SLICC-DI score cutoff of 1. And then discriminant construct validity using Mann-Whitney non-parametric tests was accessed.

### Convergent Validity

We measured the extent of correlation between observed relationships of the concepts and the hypothesized concepts to assess its convergent validity. A strong correlation was defined as ≥0.70, moderate to substantial as 0.30–0.70 and weak as <0.30.

### Factor Analysis

We conducted an exploratory factor analysis (EFA) [principal component analysis (PFA) followed by varimax rotation] with 8 imposed factor loadings consistent with the UK model to confirm its factor structure [9]. We next studied item loading on each factor after rotation, considering 0.5 as significant loading. We computed an eight-factor item loading matrix, according to the priori hypothesis of an eight-factor structure supported by the original LupusQoL dimensionality. We used Kaiser’s criterion (eigenvalues>1), Horn’s parallel analysis and graphical analysis of the screeplot to generate hypotheses about the number of factors to be extracted [16], [17].

Then we performed a confirmatory factor analysis (CFA) using the UK structure of the LupusQoL on AMOS software. In this analysis, each item was defined to represent only one domain, but the domains were allowed to correlate with each other.

A separate exploratory factor analysis (EFA) without an imposed number of domains was also performed and the model obtained was then tested by CFA.

### Rasch Analysis

Rasch analysis is the standard for the development of metric quality outcomes in healthcare [18]. We used Rasch analysis for each domain item, and obtained person and item reliability index. Misfit item was defined by the absolute value of mean square outfits >2 or <0.6 [9].

A two-tailed p value of 0.05 was considered significant in all analyses.

## Results

We first translated the traditional Chinese language of the Taiwan version of LupusQoL into “simplified” Chinese. Two experts (a rheumatologist and one Chinese linguist) made the draft versions independently. Then a multidisciplinary consensus committee was held to discuss about the availability of an agreed-on version according to the consensus of the two translators. During this meeting, the working group believed that this version was perfectly understandable and conceptual equivalence of the source. We piloted a simplified Chinese version of LupusQoL in 6 randomly selected outpatients with SLE. Feedback was sought and discussed within our working group, with the result that additional minor wording revisions were introduced; these did not result in any changes in the original meaning. The main modification was in the examples provided in the questionnaire; for instance, “ digging the garden, painting” was replaced by “carry gas canister, rice bag ” for item 1 and “dusting” was replaced by “sweeping” for item 3. The expression of “quality” used “zhiliang” instead of “pinzhi”. An additional 6 outpatients completed this modified version and no further suggestions were offered to us. This modified instrument, referred to as the LupusQoL-China, was the administered to our recruited study patients.

Among 220 SLE patients included in the study, complete data was obtained from 208. 93.8% (n = 195) were women; all were Mainland Chinese. The mean (SD) age was 33.9 (±9.19) years. The mean (SD) SLEDAI and SDI were 2.73 (±3.91) (median 2, range 0–25) and 0.37 (±0.869) (median 0, range 0–6), respectively.

The internal consistency reliability of the LupusQoL-China ranged from 0.811 to 0.965 (Table 1). The test-retest reliability ranged from 0.836 to 0.974 (Table 1). The LupusQoL-China domains had good construct validity when compared with equivalent domains on the EQ-5D (Table 2).

**Table 1:** Reliability of LupusQoL used in Chinese patients with SLE (LupusQoL-China).

Domains	Number of items	Internal consistency reliability	Test–retest reliability
Physical health	8	0.890	0.892
Pain	3	0.913	0.956
Plainning	3	0.918	0.927
Intimate relationship	2	0.965	0.880
Burden to others	3	0.931	0.974
Emotional health	6	0.961	0.970
Body image	5	0.811	0.877
Fatigue	4	0.824	0.836

**Table 2:** Convergent validity of LupusQoL used in Chinese patients with SLE (LupusQoL-China).

LupusQoL domains	EQ-5D domain	Spearman’s r
Physical health	Usual activities	−0.630
Pain	Pain/Discomfort	−0.778
Emotional health	Anxiety/depression	−0.761
Planning	Usual activities	−0.560

LupusQoL-China could discriminate patients with varied disease activities in all domains except for body image (Table 3). Similarly, it could differentiate subjects with varied disease damages in all domains except burden to others and body image (Table 4).

**Table 3:** Discriminant validity of LupusQoL used in Chinese patients with SLE (LupusQoL-China) with disease activity as the external anchor.

	SLEDAI		
Domains	0–4	>4	P value
Physical health	87.43(13.17)	57.77(25.70)	0.000
Pain	87.24(16.18)	57.58(27.59)	0.000
Planning	79.86(21.29)	53.03(28.01)	0.000
Intimate relationship	62.34(31.06)	37.93(30.16)	0.000
Burden to others	68.19(26.65)	49.49(31.73)	0.002
Emotional health	78.05(20.31)	56.19(31.27)	0.000
Body image	75.34(18.08)	72.12(15.61)	0.113
Fatigue	83.00(16.53)	68.18(21.78)	0.000

SLEDAI: SLE Disease Activity Index.

**Table 4:** Discriminant validity of LupusQoL used in Chinese patients with SLE (LupusQoL-China) with damage as the external anchor.

	SDI		
Domains	≤1	>1	P value
Physical health	87.25(12.93)	66.78(27.40)	0.000
Pain	86.47(12.59)	68.66(29.30)	0.001
Planning	79.99(21.75)	60.14(27.38)	0.000
Intimate relationship	63.10(30.56)	43.31(32.77)	0.000
Burden to others	67.59(25.99)	56.88(34.21)	0.100
Emotional health	78.22(22.40)	61.77(27.02)	0.000
Body image	75.43(18.53)	72.72(14.48)	0.109
Fatigue	82.02(16.66)	75.82(22.50)	0.025

SDI: Systemic Lupus Collaborating Clinics Damage Index.

Confirmatory factor analysis of the Chinese version of the LupusQoL using the eight domain UK version loadings of the 34 items didn’t resulted in a good fit (χ^2^/df 1.978, root mean square error of approximation (RMSEA) 0.069, goodness-of-fit index (GFI) 0.821, χ^2^ = 856.49, p = 0.001). Standardized regression weights for all items with their respective domains were >0.6, except item 1(0.54) and 33(0.57).

EFA (principal component analysis) with 8-factor imposition resulted in the finding that planning, intimate relationship, burden to others emotional health, body image and fatigue items loaded on six separate domains as expected (see Table S1). However, item 9 (pain domain) loaded on the physical health domain and the other two pain items (item 10, 11) loaded on the intimate relationship factor domain. The last item of physical health domain (item 8) loaded on one single factor. 6 of the 8 factors had an eigenvalue of >1 and cumulatively explained 74.3% of the variance. In 8 factors of the EFA result, the first factor had an eigenvalue of 14.7 and explained 43.4% of the variance. Furthermore, parallel analysis led to the retention of an 8-factor structure. Screeplot analysis suggested a 6-factor loading structure (see Table S2). Physical health and pain (item 1–11) composed the first factor. Intimate relationship and burden to others (item 15–19) constituted the same factor. Planning, emotional health, body image and fatigue had separate factor loadings. The ICR of modified six domains were as follows: first factor (physical health, pain; ICR = 0.94), second factor (planning; ICR = 0.92), third factor (intimate relationship and burden to others; ICR = 0.91), fourth factor (emotional health; ICR = 0.96), fifth factor (body image; ICR = 0.86) and sixth factor (fatigue; ICR = 0.86). The six-factor model showed similar fit statistics in confirmatory factor analysis (χ^2^ 876.05, p = 0.001, χ^2^/df 2.2, RMSEA 0.077, GFI 0.89).

For Rasch analyses, results in fit analyses of items within each domain were acceptable (mean square outfits were all >0.6 and none was >2 (Table 5). Item reliability of the pain domain was poor. Person reliability of the pain and intimate relationship domain were unsatisfactory.

**Table 5:** MNSQ values, item and person reliability indices of LupusQoL used in Chinese patients with SLE (LupusQoL-China).

Domains	Mean outfit MNSQ (SD)	Item reliability index	Person reliability index
Physical health	0.93(0.26)	0.94	0.86
Pain	0.75(0.18)	0.42	0.52
Planning	1.11(0.30)	0.98	0.89
Intimate relationship	1.09(0.34)	0.94	0.65
Burden to others	1.10(0.21)	0.98	0.80
Emotional health	1.07(0.25)	0.96	0.82
Body image	0.93(0.26)	0.90	0.71
Fatigue	1.06(0.32)	0.93	0.88

MNSQ values: Mean square outfit values, the item was considered a misfit one when its absolute value of mean square outfits >2 or <0.6.

## Discussion

Systemic lupus erythematosus, an autoimmune illness with a wide spectrum of manifestations, affects multiple organ systems and is associated with considerable morbidity and mortality. The incidence of SLE appears to be higher in China compared to America and Europe, and millions of Chinese patients are suffering from this disease [19]–[21]. Due to deeper understanding of the disease itself and its management, the disease is becoming chronic and controllable. However, because it is incurable and runs a variable course over the patient’s remaining years, SLE still profoundly affects health status, especially the HRQoL[22]–[24]. To our knowledge, there has been no SLE-specific HRQoL tools that have been validated for use among Mainland Chinese patients. Meanwhile, generic tools were not designed for SLE population, so they may contain irrelevant items and/or lack items deemed important to these patients, and may be less sensitive than a disease-specific measure [25], [26]. Accordingly, it is desirable to find an SLE-specific HRQoL tool validated for use in Chinese patients.

We modified the LupusQoL-Taiwan instrument to evaluate in Mainland Chinese patients, and assessed its measurement properties. Because there are no published data about validity and reliability of LupusQoL-Taiwan published, we compared our results with the original version (LupusQoL-UK) and LupusQoL-USA in our study [8], [9].

Our results provide evidence that the LupusQoL-China is a valid tool to assess the quality of life of SLE patients, and it is the first proposed SLE-specific HRQoL tool that may be applied to all Chinese patients.

The ICR estimates of the LupusQoL-China were similar to those of the LupusQoL-UK. The convergent and discriminant validity of the tool were good. In our data, the domain of intimate relationship which reflects sexual health can also differentiate between disease severity as well as other domains. We suggest that this result may be related to the Chinese attitude toward sexual intercourse. Traditional concepts in Chinese culture consider intercourse to be detrimental to health. When Chinese patients become ill, they tend to repress sexual desires and choose to compromise their sex life, especially in cases of severe disease.

Our exploratory factor analysis without constraining factor numbers showed a 6-factor loading model; however, the results of ICR and confirmatory factor analysis changed little. According to the description of items, we believe the original 8-factor model fits better.

To ensure the validity of test-retest reliability, we choose two weeks as the time interval between two tests, which is one week longer than in the original version of LupusQoL. This duration (two weeks) was selected because it was sufficiently long enough for patients to forget their original responses and short enough for the disease state to remain unchanged [27]. The Rasch model analysis also suggested that the LupusQoL was well-understood for our patient cohort.

It’s important to note that because the generation phase of items was conducted in the UK, which included predominantly Caucasian patients, the characteristics of Chinese patients with SLE may not be reflected completely. In addition, the difference between two country in socioeconomic status and medical care services, which may impact health status, may counter the face validity of the tool when used among Chinese SLE patients [28].

### Conclusions

In summary, our research provides evidence of validity and reliability of the modified LupusQoL version (LupusQoL-China) among Mainland Chinese patients with SLE. Additional larger studies are still required to further assess the psychometric properties and optimal factor structure, and resolve its role in clinical trials and routine practice. We hope that this study will prompt further attention to measurement of HRQoL in SLE patients, which will ultimately lead to more efficient clinical management of SLE population.

## Supporting Information

Table S1 **Exploratory Factor Analysis with 8 factor constraints.** Extraction Method: Principal Component Analysis. Rotation Method: Varimax with Kaiser Normalization.
(DOC)

Table S2 **Exploratory Factor Analysis without constraints.** Extraction Method: Principal Component Analysis. 6 factors have been extracted.
(DOCX)
