# Supplementary material for: Validity of LupusQoL-China for the Assessment of Health Related Quality of Life in Chinese Patients with Systemic Lupus Erythematosus
Source: PLoS One. 2013 May 23;8(5):e63795. doi: 10.1371/journal.pone.0063795 (PMC3662722; doi:10.1371/journal.pone.0063795)
Supplement: Table S1 — Exploratory Factor Analysis with 8 factor constraints. Extraction Method: Principal Component Analysis. Rotation Method: Varimax with Kaiser Normalization. (DOC) [file pone.0063795.s001.doc]

Table S1: Exploratory Factor Analysis with 8 factor constraints.

Rotated Component Matrix(a)

|  | Component | | | | | | | |
| --- | --- | --- | --- | --- | --- | --- | --- | --- |
|  | 1 | 2 | 3 | 4 | 5 | 6 | 7 | 8 |
| Physical Health 1 | .322 | **.659** | .150 | .165 | .093 | .142 | .004 | .240 |
| Physical Health 2 | .321 | **.756** | .162 | .162 | .075 | .148 | .157 | .144 |
| Physical Health 3 | .185 | **.818** | .026 | .159 | .132 | .182 | .119 | -.111 |
| Physical Health 4 | .118 | **.741** | .063 | .112 | .284 | .161 | .153 | .080 |
| Physical Health 5 | .283 | **.673** | .076 | -.055 | .298 | .140 | .278 | .178 |
| Physical Health 6 | .250 | **.635** | .068 | .115 | .384 | .205 | .241 | .186 |
| Physical Health 7 | .190 | **.582** | .023 | .143 | .035 | .214 | .136 | .543 |
| Physical Health 8 | .153 | .223 | .069 | .134 | .179 | .214 | .235 | **.755** |
| Pain 1 | .365 | .**501** | .180 | .128 | .325 | .133 | .011 | .469 |
| Pain 2 | .350 | .330 | .191 | -.083 | **.593** | .362 | .052 | .275 |
| Pain 3 | .262 | .337 | .150 | -.026 | **.679** | .250 | .073 | .349 |
| Planning 1 | .185 | .332 | .158 | .341 | .173 | .274 | **.637** | .154 |
| Planning 2 | .313 | .386 | .208 | .105 | .110 | .178 | **.680** | .174 |
| Planning 3 | .262 | .408 | .253 | .205 | .168 | .069 | **.663** | .182 |
| Int. Relationship 1 | -.008 | .216 | .032 | .371 | **.833** | -.019 | .074 | .015 |
| Int. Relationship 2 | .004 | .205 | .002 | .369 | **.833** | -.023 | .114 | -.004 |
| Burden to others 1 | .146 | .172 | .134 | **.768** | .246 | .315 | .204 | -.088 |
| Burden to others 2 | .217 | .165 | .106 | **.870** | .121 | .229 | .064 | .121 |
| Burden to others 3 | .165 | .123 | .155 | **.811** | .290 | .098 | .159 | .186 |
| Emotional Health 1 | **.819** | .145 | .117 | .246 | .039 | .138 | .004 | .259 |
| Emotional Health 2 | **.859** | .258 | .131 | .057 | .096 | .122 | .041 | .131 |
| Emotional Health 3 | **.900** | .227 | .112 | .115 | .063 | .049 | .062 | .102 |
| Emotional Health 4 | **.860** | .203 | .103 | .130 | .051 | .134 | .136 | .186 |
| Emotional Health 5 | **.826** | .197 | .168 | .078 | .040 | .233 | .252 | -.129 |
| Emotional Health 6 | **.819** | .195 | .172 | .078 | .118 | .173 | .214 | -.071 |
| Body Image 1 | .217 | .024 | **.687** | .229 | .019 | .077 | .308 | -.095 |
| Body Image 2 | .104 | -.077 | **.784** | .104 | -.011 | .090 | .298 | .029 |
| Body Image 3 | .253 | .047 | **.828** | -.007 | .092 | .094 | .160 | -.004 |
| Body Image 4 | -.029 | .311 | **.688** | -.058 | .093 | .029 | -.190 | .079 |
| Body Image 5 | .129 | .127 | **.780** | .165 | .034 | .003 | -.063 | .139 |
| Fatigue 1 | .297 | .282 | .195 | .376 | .250 | **.556** | -.092 | .151 |
| Fatigue 2 | .316 | .218 | .148 | .180 | .166 | **.748** | .131 | .151 |
| Fatigue 3 | .144 | .092 | .111 | .305 | -.009 | **.615** | .318 | .045 |
| Fatigue 4 | .128 | .293 | -.029 | .101 | -.003 | **.749** | .042 | .124 |

Extraction Method: Principal Component Analysis.

Rotation Method: Varimax with Kaiser Normalization.
