# Supplementary material for: Validity of LupusQoL-China for the Assessment of Health Related Quality of Life in Chinese Patients with Systemic Lupus Erythematosus
Source: PLoS One. 2013 May 23;8(5):e63795. doi: 10.1371/journal.pone.0063795 (PMC3662722; doi:10.1371/journal.pone.0063795)
Supplement: Table S2 — Exploratory Factor Analysis without constraints. Extraction Method: Principal Component Analysis. 6 factors have been extracted. (DOCX) [file pone.0063795.s002.docx]

Table S2: Exploratory Factor Analysis without constraints

|  | Component | | | | | |
| --- | --- | --- | --- | --- | --- | --- |
|  | 1 | 2 | 3 | 4 | 5 | 6 |
| Physical Health 1 | **.654** | .307 | .130 | .093 | .206 | .046 |
| Physical Health 2 | **.719** | .307 | .143 | .076 | .187 | .222 |
| Physical Health 3 | **.692** | .174 | -.006 | .127 | .147 | .260 |
| Physical Health 4 | **.755** | .100 | .049 | .208 | .117 | .166 |
| Physical Health 5 | **.798** | .258 | .079 | .109 | .036 | .174 |
| Physical Health 6 | **.762** | .231 | .072 | .276 | .154 | .152 |
| Physical Health 7 | **.710** | .171 | .026 | .014 | .332 | .060 |
| Physical Health 8 | **.552** | .135 | .109 | .124 | .349 | -.027 |
| Pain 1 | **.688** | .344 | .184 | .237 | .182 | -.121 |
| Pain 2 | **.652** | .320 | .211 | .290 | .177 | -.231 |
| Pain 3 | **.681** | .233 | .175 | .405 | .108 | -.224 |
| Planning 1 | .454 | .187 | .191 | .271 | .353 | **.553** |
| Planning 2 | .536 | .305 | .242 | .080 | .177 | **.551** |
| Planning 3 | .532 | .258 | .284 | .202 | .123 | **.567** |
| Int. Relationship 1 | .371 | -.009 | .047 | **.850** | -.028 | -.032 |
| Int. Relationship 2 | .363 | .003 | .019 | **.851** | -.038 | .005 |
| Burden to others 1 | .101 | .170 | .135 | **.618** | .536 | .334 |
| Burden to others 2 | .074 | .244 | .101 | **.590** | .587 | .232 |
| Burden to others 3 | .123 | .189 | .165 | **.697** | .428 | .236 |
| Emotional Health 1 | .221 | **.818** | .120 | .124 | .264 | -.025 |
| Emotional Health 2 | .335 | **.850** | .129 | .048 | .119 | -.007 |
| Emotional Health 3 | .269 | **.896** | .110 | .074 | .086 | .049 |
| Emotional Health 4 | .295 | **.855** | .111 | .059 | .183 | .079 |
| Emotional Health 5 | .219 | **.824** | .171 | .022 | .172 | .240 |
| Emotional Health 6 | .247 | **.816** | .177 | .086 | .120 | .177 |
| Emotional Health 7 | .010 | .228 | **.697** | .139 | .134 | .322 |
| Body Image 1 | -.014 | .110 | **.804** | .035 | .123 | .231 |
| Body Image 2 | .118 | .249 | **.836** | .032 | .044 | .080 |
| Body Image 3 | .301 | -.042 | **.665** | -.003 | -.010 | -.184 |
| Body Image 5 | .124 | .129 | **.771** | .102 | .098 | -.049 |
| Fatigue 1 | .361 | .292 | .188 | .310 | **.607** | -.116 |
| Fatigue 2 | .391 | .305 | .160 | .096 | **.688** | .000 |
| Fatigue 3 | .168 | .148 | .129 | .083 | **.651** | .274 |
| Fatigue4 | .385 | .115 | -.030 | -.070 | **.685** | -.020 |

Extraction Method: Principal Component Analysis. 6 factors have been extracted.
